# Metabolic Changes in Maternal and Cord Blood in One Case of Pregnancy-Associated Breast Cancer Seen by Fluorescence Lifetime Imaging Microscopy

**DOI:** 10.3390/diagnostics11081494

**Published:** 2021-08-19

**Authors:** Li Zhou, Yawei Kong, Junxin Wu, Xingzhi Li, Yiyan Fei, Jiong Ma, Yulan Wang, Lan Mi

**Affiliations:** 1 Shanghai Engineering Research Center of Ultra-Precision Optical Manufacturing, Key Laboratory of Micro and Nano Photonic Structures (Ministry of Education), Department of Optical Science and Engineering, School of Information Science and Technology, Fudan University, 220 Handan Road, Shanghai 200433, China; zhoul16@fudan.edu.cn (L.Z.); 18110720005@fudan.edu.cn (Y.K.); 18210720098@fudan.edu.cn (J.W.); fyy@fudan.edu.cn (Y.F.); jiongma@fudan.edu.cn (J.M.); 2 Department of Biochemistry and Molecular Biology, School of Life Sciences, Fudan University, 220 Handan Road, Shanghai 200433, China; 3 School of Materials Science and Engineering, Hubei University of Automotive Technology, 167 Checheng West Road, Shiyan 442002, China; 20010037@huat.edu.cn; 4 Institute of Biomedical Engineering and Technology, Academy for Engineer and Technology, Fudan University, 220 Handan Road, Shanghai 200433, China; 5 Shanghai Engineering Research Center of Industrial Microorganisms, The Multiscale Research Institute of Complex Systems (MRICS), School of Life Sciences, Fudan University, 220 Handan Road, Shanghai 200433, China; 6 Department of Gynecology and Obstetrics, The Central Hospital of Wuhan, Tongji Medical College, Huazhong University of Science and Technology, 26 Shengli Str., Wuhan 430014, China

**Keywords:** pregnancy-associated breast cancer, blood, fluorescence lifetime imaging microscopy, NAD(P)H, FAD

## Abstract

Pregnancy-associated breast cancer (PABC) is a rare disease, which is frequently diagnosed at an advanced stage due to limitations in current diagnostic methods. In this study, fluorescence lifetime imaging microscopy (FLIM) was used to study the metabolic changes by measuring maternal blood and umbilical cord blood via the autofluorescence of coenzymes, reduced nicotinamide adenine dinucleotide (phosphate) (NAD(P)H), and flavin adenine dinucleotide (FAD). The NAD(P)H data showed that a PABC case had significant differences compared with normal cases, which may indicate increased glycolysis. The FAD data showed that both maternal and cord blood of PABC had shorter mean lifetimes and higher bound-FAD ratios. The significant differences suggested that FLIM testing of blood samples may be a potential method to assist in PABC non-radiative screening.

## 1. Introduction

According to the updated global cancer statistics 2020, breast cancer is one of the most frequent malignancies and the leading cause of cancer death in females worldwide [1]. The clinical situation can be more complex with pregnancy-associated breast cancer (PABC), which is defined as breast cancer diagnosed during pregnancy or within the first 12 months postpartum. The overall incidence of PABC is low—about 6.5 cases per 100,000 births [2]. PABC is one of the most serious threats to pregnant women; however, the extremely low probability may lead to negligence in self-detection and clinical examination. Moreover, normal physiological changes in the breast, such as engorgement and tenderness, frequently happen during pregnancy, which increases the difficulty of diagnosis of PABC by physical imaging. Ultrasound and mammography are recommended to evaluate a breast mass. Even though ultrasound has a sensitivity close to 100%, it brings a high negative prediction rate [3]. Mammography is used for further detection and characterization, but research has shown that it is less sensitive, detecting 86.7% of cases of PABC, and it poses a radiation risk to fetus and pregnant patient [4]. In another cohort study, women below 40 years old who underwent PABC had more aggressive subtypes, such as triple-negative breast cancer up to 5 years after parturition [5]. However, with more women delaying childbearing for personal and professional reasons, the prevalence of PABC may rise in the future. Due to the limitations of current imaging methods of screening for PABC, it is essential to develop new diagnostic methods.

It is well known that nicotinamide adenine dinucleotide (phosphate) (NAD(P)H) and flavin adenine dinucleotide (FAD) act as electron carriers and take part in a variety of biochemical reactions, particularly the tricarboxylic acid cycle and oxidative phosphorylation [6,7]. The intrinsic fluorescence properties of NAD(P)H and FAD can be used to monitor metabolic status at the cellular level by fluorescence lifetime imaging microscopy (FLIM) without any sophisticated label [8]. It is known that the fluorescence lifetimes of NAD(P)H and FAD depend on the binding to proteins. Free NAD(P)H has a short fluorescence lifetime of about 0.3—0.6 ns, while bound NAD(P)H has increased fluorescence lifetime of 1—4 ns. The ratio of free and bound NAD(P)H is related with the types of the metabolism. For FAD, unbound FAD has a lifetime of a few ns, and bound FAD of a few 100 ps. FLIM is independent of fluorophore concentration, excitation intensity, and optical path length, which allows the visualization of fluorophore lifetime, as well as its interaction with the biological environment. FLIM has been used to study the difference between precancerous and cancerous cells [7] and to detect various malignancies in unstained human tissues [8,9,10,11,12]. As an important medium circulating in the whole body, blood carries a lot of information about infection, brain injuries, and cancers [13], including protein markers and circulating tumor DNA. The fluorescence spectroscopy of blood plasma has been reported as fingerprints of glucose metabolism in a rat model [14]. So far, there is limited information on the metabolic state of PABC patients’ blood samples. In this study, we studied blood samples from a PABC patient and compared them with the normal cases by label-free FLIM, as well as placental tissues, to display the difference of the metabolic status between PABC and normal cases.

## 2. Materials and Methods

### 2.1. Sample Preparation

This study was approved by the Institutional Ethic Committee of the Central Hospital of Wuhan, Tongji Medical College, Huazhong University of Science and Technology. Blood and placenta tissue samples were provided by the Central Hospital of Wuhan. The samples included maternal blood, umbilical cord blood, and placental tissue sections from one PABC and seven normal parturients, whose age was between 26 and 29 years old. All the neonates had Apgar scores of 10. The PABC case in this study was not treated for breast cancer before delivery. The hematocrit and blood cell counts did not show difference for all the cases. Red blood cells (RBCs) are approximately 7.5 μm in diameter and with average concentration of 3.5–6.0 × 10^6^/μL. White blood cells (WBCs) are classified into five types, in which neutrophils are the predominant WBCs in adults and with a diameter of 7–18 μm [15]. The normal concentration of WBCs is approximately 3.6–10 × 10^3^/μL, much lower than that of RBCs. Platelets are small and irregular cells with a diameter of 2-4 μm and normal concentration is between 1 × 10^5^/μL and 3 × 10^5^/μL.

Maternal blood collection was conducted from the antecubital vein after overnight fasting with vacuum blood tubes containing ethylenediaminetetraacetic acid (EDTA) dipotassium salt before delivery, and cord blood samples were collected from the umbilical cord with vacuum blood tubes after delivery. All the maternal and umbilical cord blood samples were kept at 4 °C before observation during transportation within three days for the PABC case and the FLIM laboratory are in two cities. About 3 μL of blood was smeared on glass slides for the FLIM observation. In order to study the influence of time on the autofluorescence of blood, fresh antecubital vein blood samples from eight patients without malignant tumors were collected at the local hospital and studied every day for 7 days. The samples were stored at 4 °C. 

The placental tissue samples were freshly collected after cesarean delivery as the conventional protocol. Briefly, tissues were placed into 10% formalin for fixation, processed with ethanol and xylene (70% ethanol for 1 h, 95% ethanol for 1 h, 100% ethanol for 1 h, xylene for 100 min twice), and then placed in paraffin wax at 60 °C for 90 min 2 times. The paraffin-embedded samples were cut into slices of 4 µm thickness and placed on glass without staining for the FLIM study.

### 2.2. FLIM Setup

**Figure 1:**
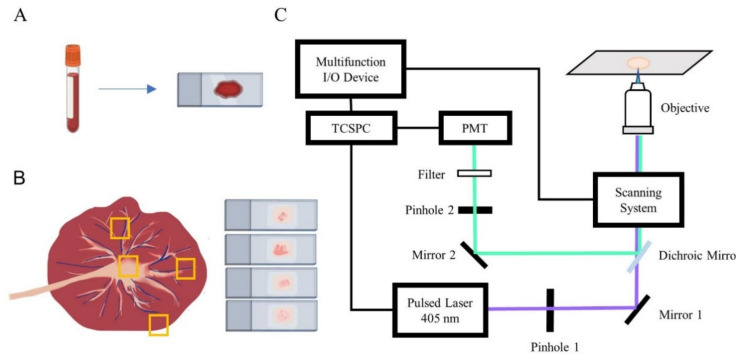
Diagram of sample preparation and equipment. (**A**) Preparation of blood glass smear. (**B**) Schematic diagram of placental sampling at various sites as marked by the yellow boxes. (**C**) FLIM setup.

Blood samples were excited by a 405 nm picosecond laser (BDL-405-SMC, Becker & Hickl, Berlin, Germany) and imaged with a confocal laser scanning microscope (FV300/IX71, Olympus, Japan) and a time correlated single photon counting (TCSPC) system (SPC-150, Becker & Hickl, Germany), with a 60× water-dipping objective (NA = 1.2). The schematic design of the FLIM optical system is shown in Figure 1. Autofluorescence signal was collected by a photomultiplier tube (PMC-100-1, Becker & Hickl, Germany) with a 417–477 nm bandpass filter for NAD(P)H and a 532 nm long-pass filter for FAD. Each FLIM image has 256 × 256 pixels, and each sample was measured by FLIM for NAD(P)H at 5-6 different areas and for FAD at other 5–6 areas. Fluorescence lifetime imaging of placental tissue was acquired using Leica TCS SP8 DIVE FALCON (Leica, Berlin, Germany), equipped with a 63× water-dipping objective (NA = 1.2) and excited by a femtosecond pulsed laser of 810 nm (InSight X3 Dual, Spectra-Physics, Santa Clara, CA, USA). The emission window was set as 417–477nm for NAD(P)H and 532–632 nm for FAD.

According to the excitation spectra of NAD(P)H and FAD, the 375 nm lasers are found reasonable for excitation. However, scanners of most confocal laser scanning microscopes and microscope objectives are not designed for these wavelengths. Therefore, the 405 nm laser was used instead. For instance, Madhuri et al. observed an emission peak around 460 nm of NAD(P)H with 405 nm excitation for human blood plasma [16]. Bhatta et al. excited yeast cells with 405 nm and observed the autofluorescence of flavins compounds [17]. Becker et al. reported FLIM of FAD with 405 nm excitation and 490 to 600 nm emission filter [18].

### 2.3. Image and Data Processing

The fluorescence lifetime data obtained by the Olympus microscope was analyzed by a double-exponential decay fitting method with the software SPCImage (Becker & Hickl, Germany). The FLIM data obtained by the Leica microscope was fitted with the software LAS X(Leica Microsystems, Berlin, Germany). 

The mean fluorescence lifetime was calculated as:

*t*_m_ = *a*_1_*t*_1_ + *a*_2_*t*_2_, (1)

where *t*_m_ is the mean lifetime, *t*_1,2_ are two lifetime components, and *a*_1,2_ are the proportion of the components. For NAD(P)H, free NAD(P)H has a short lifetime, as previously reported and measured by a free NADH solution, *t*_1_, around 460 ps [19]; and bound NAD(P)H has a long lifetime, *t*_2_, ranging between 1.0 and 5.7 ns, when binding to different proteins [20]. For FAD, bound FAD has a short lifetime, *t*_1_, of a few hundred ps, while free FAD has a longer lifetime of *t*_2_ around 2730 ps [21]. When fitting with a double exponential decay, *t*_1_ of NAD(P)H and *t*_2_ of FAD were fixed, and then the *t*_m_ and *a*_1,2_ of all pixels were calculated. The *t*_m_ or *a*_1,2_ distribution curve of the 256 × 256 pixels in each FLIM image can be obtained by the SPCImage software (SPCImage 8.0, Becker & Hickl, Germany). The multi-peaks of the lifetime distribution curve are fitted using a Gaussian function for each FLIM image, and at least five images are studied for each sample and averaged for statistics.

## 3. Results

### 3.1. FLIM of NAD(P)H in Blood

**Figure 2:**
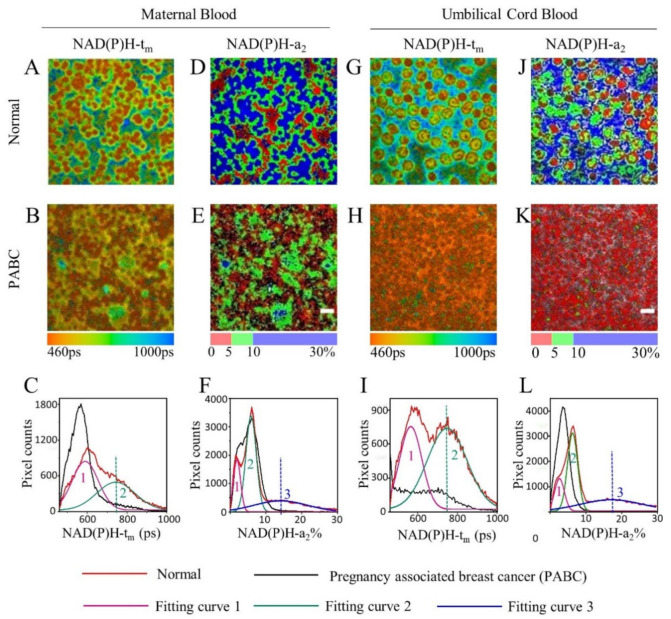
Representative NAD(P)H FLIM images and their distribution curves of normal and PABC cases. (**A**,**B**,**D**,**E**) *t*_m_ and *a*_2_ images of maternal blood, (**G**,**H**,**J**,**K**) *t*_m_ and *a*_2_ images of umbilical cord blood. (**C**,**F**,**I**,**L**) Distribution curves of corresponding images. Scale bar: 10 μm.

For the NAD(P)H FLIM study, a representative mean lifetime (*t*_m_) image of normal maternal blood (Figure 2A) demonstrated the blood cells in orange, and plasma in green and blue, indicating a longer lifetime in plasma than in blood cells. The situation of PABC maternal blood was different (Figure 2B), and mainly displayed orange and yellow, representing a shorter lifetime than that in the normal group. Figure 2C shows the *t*_m_ distribution curves of Figure 2A,B. The normal curve had a relatively wider range than that of PABC. For the normal curve, two peaks can be fitted as peak 1 at 589 ps and peak 2 at 743 ps. It is noticed that peak 1 is close to the distribution of the PABC curve, with only one peak at 568 ps. The height of peak 2 in the normal group is about 8.7 times of that from PABC (Figure 2C). Figure 2D,E display the percentage of bound NAD(P)H, indicated as *a*_2_, for normal and PABC. The blood cells shown in red had *a*_2_ less than 5%, and the plasma shown in green and blue had *a*_2_ values ranging from 5% to 30% for the normal sample. For PABC, the plasma’s *a*_2_ was mostly in green (5–10%), which is significantly lower than that of normal. The distribution curves of NAD(P)H-*a*_2_ (Figure 2F) showed three peaks for the normal sample, around 2%, 6%, and 14%. However, the distribution curve of PABC had two peaks (2% and 6%). The significant difference between PABC and normal was that the normal curve had a broad peak at 14%, while PABC had little distribution there (peak 3 in Figure 2F). This may partly explain why the normal NAD(P)H-*t*_m_ is relatively wider than that of PABC. The NAD(P)H FLIM images and distribution curves of umbilical cord blood samples were similar to the corresponding maternal blood results (Figure 2G–L).

**Figure 3:**
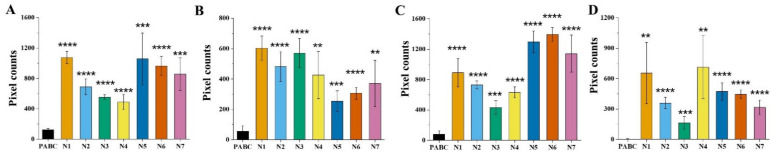
The average NAD(P)H *t*_m_ and *a*_2_ distribution curve peak heights of PABC and the normal group (including seven pairs of normal maternal blood and umbilical cord blood, N1-N7). (**A**) NAD(P)H-*t*_m_ peak 2 height in maternal blood, (**B**) NAD(P)H-*a*_2_ peak 3 height in maternal blood, (**C**) NAD(P)H-*t*_m_ peak 2 height in cord blood, and (**D**) NAD(P)H-*a*_2_ peak 3 height in cord blood (** *p* <0.01, *** *p* <0.001, **** *p* <0.0001).

Figure 3 shows the statistical data of the NAD(P)H FLIM of blood, including one PABC case and seven normal cases (noted as N1–N7). The heights of peak 2 marked in Figure 2C,I and the heights of peak 3 in Figure 2F,L were analyzed and averaged from five FLIM images for each sample. It was found that the values of the normal group were significantly higher than that of PABC. Although there was individual variation among the seven normal cases, the average heights of peak 2 of the *t*_m_ curves were 6.6 ± 1.8 times and 11.9 ± 4.3 times that of PABC for maternal blood and cord blood, respectively. Meanwhile, the average heights of peak 3 of the *a*_2_ curves for the normal group were 7.6 ± 2.4 and 144.1 ± 57.2 times higher than that of PABC for maternal and cord blood, respectively.

The PABC case had a lower NAD(P)H *t*_m_ and *a*_2_, indicating that the metabolic pathway may have a change towards glycolysis [22]. This result is consistent with a number of studies in cultured cancer cells [23] and in cancer tissues [10,11,24]. The similar characteristics in Figure 2F,L indicate that the metabolic status of cord blood and corresponding maternal blood is similar for both normal women and the patient with PABC. It suggested that metabolic status of cord blood was correlated with that of maternal blood. As reported in a previous study [25], placental metastases may occur. In this study, the PABC placenta looked histologically normal (Figure S1) as normal cases, and no detectable metabolic difference in the placenta was found between the PABC case and normal cases. Although no metastasis was observed in the placenta for this PABC case, the cord blood from the PABC case may be affected by abnormal maternal metabolism through the placenta.

### 3.2. FLIM of FAD in Blood

**Figure 4:**
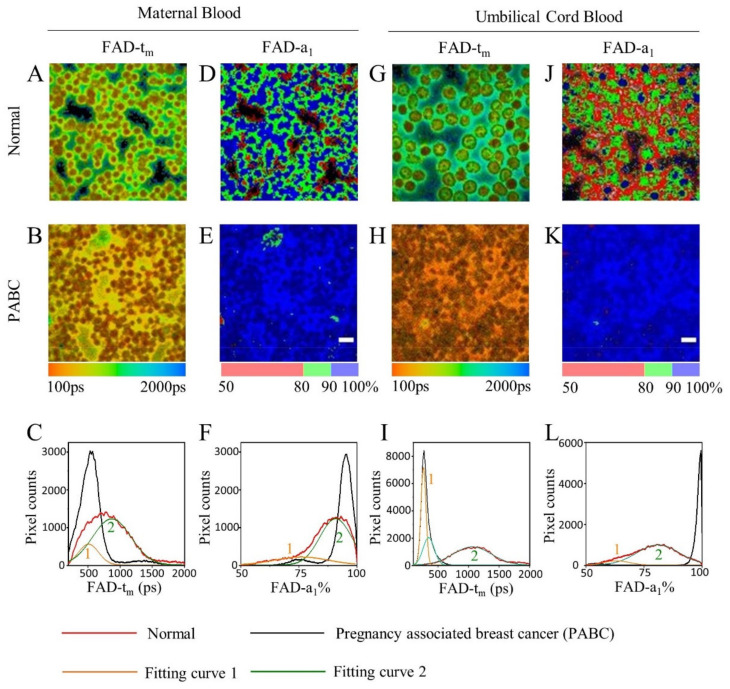
Representative FAD FLIM images and their distribution curves of PABC and normal cases. (**A**,**B**,**D**,**E**) *t*_m_ and *a*_1_ images of maternal blood, (**G**,**H**,**J**,**K**) *t*_m_ and *a*_1_ images of umbilical cord blood. (**C**,**F**,**I**,**L**) Distribution curves of corresponding images. Scale bar: 10 μm.

FAD is also an important coenzyme in metabolism. Representative FAD mean lifetime (*t*_m_) and percentage of bound-FAD (*a*_1_) images of PABC and normal blood samples were compared (Figure 4). The FAD-*t*_m_ of maternal blood in PABC is shorter than that of normal, especially in plasma (Figure 4A,B). Figure 4C displays the *t*_m_ distribution curves of Figure 4A,B. PABC had a single narrow peak at 557 ps, which overlapped with peak 1 of the normal curve, but was nearly absent at peak 2 of normal curve (Figure 4C). 

Peak 2 in normal cases may originate from the fluorescence in plasma. Figure 4D shows that FAD-*a*_1_ of normal maternal blood cells was higher than 90%, while that of plasma decreased to 50–90%. In contrast, for PABC, FAD-*a*_1_ values of both blood cells and plasma were more than 90% (Figure 4E). The *a*_1_ distribution curve demonstrated that the full width at half maximum (FWHM) of the normal maternal blood group is 2.8 times that in the PABC case (Figure 4F). This indicates that there is a difference in FAD status between blood cells and plasma in normal maternal blood, while the FAD status in the PABC blood sample has undergone major changes. With the bound-FAD ratio increased in PABC, the FAD-*t*_m_ was slightly smaller, and the distribution was narrower. The FAD images and distribution characteristics of umbilical cord blood were similar to those of maternal blood, as shown in Figure 4G–L.

**Figure 5:**
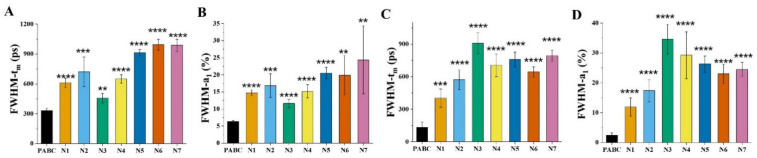
The FWHM of the FAD *t*_m_ and *a*_1_ distribution curves of PABC and the normal group (including seven pairs of normal maternal blood and umbilical cord blood, N1-N7). (**A**) FAD-*t*_m_ of maternal blood, (**B**) FAD-*a*_1_ of maternal blood, (**C**) FAD-*t*_m_ of cord blood, (**D**) FAD-*a*_1_ of cord blood (** *p* < 0.01, *** *p* < 0.001, **** *p* < 0.0001).

Statistical analyses of the FWHM of the peaks in Figure 4C–L were performed, as well as of the other FLIM images. At least five FLIM images of each sample were analyzed, and the averaged FWHM values are displayed in Figure 5. The maternal blood distributions of FAD-*t*_m_ and FAD-*a*_1_ in the normal group were significantly wider than those in the PABC case (*p* < 0.01), and their FWHMs were 2.3 ± 0.6 times and 2.8 ± 0.6 times that from PABC, respectively. The gap in cord blood samples was even greater. The FWHM of normal cord blood *t*_m_ and *a*_1_ were 5.0 ± 1.1 times and 9.7 ± 2.8 times that of PABC, respectively.

The FAD data of blood showed that both maternal and cord blood of PABC had shorter mean lifetimes and higher bound-FAD ratios. The metabolic states in plasma and blood cells of PABC were similar, so the distribution was more concentrated than normal cases.

The influence of time on the autofluorescence of blood samples from eight patients without malignant tumors were studied over seven days. Figure S2 presented an example of one antecubital vein blood sample, and Figure S2A,B showed a group of *t*_m_ and *a*_2_ distribution curves of NAD(P)H FLIM images within seven days. There are small changes among the curves from fresh samples to stored for seven days. All the other seven blood samples are similar with typical curves in Figure S2. Since the statistical analysis (Figure 3) was based on the height of NAD(P)H-*t*_m_ peak 2 and NAD(P)H-*a*_2_ peak 3 in Figure 2, the height of corresponding peaks in Figure S2A,B were analysed as well and the averaged values were displayed in Figure S2C,D. For comparison, the peak height of the PABC case was also shown. It can be seen that all the peak heights of the control group (tumor-free patients) stored for 0–6 days were much higher than the results of PABC. Similar results can be found for FAD in Figure S3. Therefore, the influence of time on the NAD(P)H and FAD fluorescence of blood is negligible in this work.

## 4. Discussion

PABC is a rare disease. As reported in a population-based cohort study on 8.8 million births, an overall 10-year incidence of PABC is 6.5 cases per 100,000 births [2]. This rare case has only been found once in the hospital where the authors work for decades. Therefore, the cohort in this study is small. Fortunately, for this PABC patient, there are two blood samples—maternal blood and umbilical cord blood. The umbilical cord blood samples showed similar metabolic status results, compared to the corresponding maternal blood results, whether it is from the PABC patient or from the normal group. It suggested that metabolic status of cord blood was correlated with that of maternal blood. Nevertheless, more PABC cases’ study would be useful to clarify the differences between PABC and normal cases.

Despite extensive researches on FLIM in the medical field to characterize cellular and tissular metabolic changes for cancer, only a handful of published studies have reported on the NAD(P)H and FAD in blood for cancer detection. Kalaivani et al. found a difference in blood obtained from cervical cancer patients and normal controls by time-resolved spectra [24]. Organs can interact with the blood circulation. It was reported that NAD (NAD^+^ and NADH) concentrations decreased both in blood and tissues (including liver, kidney, stomach, cerebrum, lung, spleen, and large and small intestine) when mice were fed a NiA-free diet [26]. It was also reported that NADH together with SIRT protein promote tumorigenesis [27]. It could be assumed that PABC disease influences NAD(P)H in blood as well.

Blood is composed of a variety of components, including RBCs, WBCs, platelets, and plasma. According to the size of blood cells and their average concentrations, most of the blood cells shown in Figure 2 and Figure 4 are RBCs. As presented in Figure S4, a few cells were observed with larger size and different fluorescence lifetimes than RBCs, which might be WBCs. The metabolism of RBCs indirectly mirrors systemic metabolic status as they circulate in the bloodstream, owing to the abundance of metabolite transporters in RBC membranes [28]. The metabolites in RBCs involved in glycolysis and Krebs cycle were studied, and some of them were found to be a signal and mediator in the whole organism, including muscles, liver, brain, and cancer cells [29]. The NAD concentration was reported in the range of 10–60 μM in human RBCs [30], while the concentration in blood plasma varies in different reports [31,32]. Therefore, both intracellular and extracellular NAD(P)H of blood samples should be studied for metabolism. In the present study, whole blood with a small amount was tested for each sample. No centrifugation operation was needed and the autofluorescence from both blood cells and blood plasma was detected. The NAD(P)H mean lifetime (*t*_m_) of PABC blood was shorter than that of normal blood (Figure 2C). The bound-NAD(P)H proportion (*a*_2_) of PABC blood was significantly lower than that of normal (Figure 2F). Similar results have been reported in many cancer tissues, such as breast cancer [8,20,33], cervical cancer [10,24], and lung cancer [11]. Most reports have suggested that this phenomenon was caused by the Warburg effect [34]; that is, cell carcinogenesis promoted a metabolic shift to glycolysis, with oxidative phosphorylation inhibited, thus reducing the fluorescence lifetime of NAD(P)H and the proportion of bound NAD(P)H. In order to meet the need for rapid synthesis of nucleic acids and fatty acids and maintain a reduced state in fast-dividing cancer cells, some changes may occur in the metabolic pathways of cancer cells, including increasing the pentose phosphate pathway (PPP) [35]. The PPP specifically produces NADPH, which leads to an increase in the free NADPH pool and a decrease in the proportion of bound NADPH. In addition, immune cells and tumor cells are in a tumor microenvironment and have similar physical stresses, glycolysis metabolism, and the Warburg effect are also found valid for myeloid cells and lymphoid cells, i.e., macrophages, and mainly for circulating lymphocytes, even more than for RBCs [36]. It was reported that tumor acidity and altered nutrient availability affected the metabolic reprogramming of T lymphocytes, resulting in differentiation towards suppressive phenotypes [37]. The metabolic profiles of cancer cells and activated T lymphocytes are remarkably similar [37]. Macrophage activity against pathogens and tumor cells requires aerobic glycolysis, which is required for both the M1 and M2 polarization state of macrophages [38]. Glucose levels in the tumor microenvironment due to high glucose consumption alter the activation status of tumor-associated macrophages and drive the metabolic changes and a switch to glycolytic metabolism in macrophages [39]. Thus, the role of a variety of blood cells should be considered to study the metabolic changes.

FAD is another redox cofactor mainly localized in mitochondria, also some in the nuclei and cytosolic compartment; besides, the HPLC experiments showed that rat liver nuclei contain protein-bound FAD [40]. Even though RBCs do not have nuclear or mitochondria, they still contain FAD. The FAD mean lifetime (*t*_m_) of maternal and cord blood from PABC is shorter than that from the normal group, and the proportion of bound-FAD (*a*_2_) in PABC blood is higher than that of the normal group (Figure 4). This result is consistent with our previous report of human lung cancer tissues and cultured human bronchial epithelial cells (BEAS-2B) [11]. That is, the fluorescence lifetime of human lung cancer tissue was shorter than that of normal lung tissue, and the fluorescence lifetimes of both NAD(P)H and FAD in BEAS-2B cells decreased when inhibiting cell oxidative phosphorylation. As previously reported [29], RBCs can be impacted by systemic metabolic homeostasis and other cell functions, and are found involved in inflammation, neurodegenerative diseases, aging, and cancer. As discussed above, other blood cells as macrophages and lymphocytes can also reflect the metabolic changes of tumor microenvironment and are impacted by metabolic changes, even more than RBCs. We hypothesize that the metabolic change of PABC would indirectly response in the variety of blood cells and plasma. The FAD results (Figure 4 and Figure 5) indicated that the glycolysis pathway may be more dominant in the PABC case due to the Warburg effect, resulting in the decrease of the fluorescence lifetime of NAD(P)H and FAD, either in cancer cells or in blood. It has been reported that the riboflavin levels, the coenzyme activation of erythrocyte glutathione reductase [41], fructose and glucose concentrations [42], glucose and glycosylated haemoglobin levels [43] in maternal blood and cord blood showed significant correlation. In this work, the FAD difference both in maternal blood and cord blood might be caused by the correlation between them as well. However, the FAD result is different from a few reports on cancer tissues or cancer cells. For example, an increase in FAD lifetime was observed in highly cancerous epithelial cells in a hamster oral model [44] and in human pancreatic cancer tissues [45] than in normal cells or tissues. Since the microenvironment of a tumor is quite complicated, the changes of the microcirculation and the partial oxygen pressure will vary greatly with the location of the cancer cells and the cancer development progress. The metabolic pathway of tumor cells can shift between glycolysis and oxidative phosphorylation [46,47]. Therefore, it is possible to find different trends of the FAD fluorescence lifetime in different cancer diseases.

Although maternal cancer during pregnancy is rare, metastatic maternal malignancy to the placenta and/or fetus have been reported more than 100 cases in recent decades [48]. Froehlich et al. reported a case of PABC with the large amount of placenta metastases, and cancer cells were observed only in the placental intervillous space, but not in the villi or other extraembryonic fetal tissues [25]. Miller et al. reported a case of rapidly progressive and fatal gastric carcinoma during pregnancy, with spread to the maternal blood space within the placenta but no chorionic villous invasion [48]. It is likely that placental metastases are more common than reported, especially with a lack of or inadequate pathologic examination of placentas [49]. In this study, the PABC placenta looked histologically normal (Figure S1), and no detectable difference in the placenta was found between the PABC and normal cases by two-photon excitation FLIM. The major advantage of two-photon excitation is the greater depth of tissue penetration compared with the single-photon excitation. For tissue study, especially those thick tissue that cannot be sliced, two-photon excitation is more appropriate. Considering the potential placental and fetal metastasis, it is of importance to examine the placenta carefully for pregnancy-related cancer even if macroscopic inspection is normal.

## 5. Conclusions

In summary, this study performed a FLIM study to detect metabolic status by NAD(P)H and FAD fluorescence in maternal blood and cord blood from a PABC case, and compared with seven normal cases. It was found that the metabolic status of maternal blood and the corresponding cord blood were similar for either the PABC case or the normal group. More importantly, the mean lifetimes of NAD(P)H and FAD in PABC blood were lower than in the normal group, and the FAD distribution range of PABC was much narrower than that of the normal group. The results of the reported PABC case suggest the use of FLIM testing to identify metabolic changes in PABC and support further analysis in a larger sample size.

## Data Availability

Data are available from the corresponding author upon reasonable request.

## Supplementary Materials

The following are available online at https://www.mdpi.com/article/10.3390/diagnostics11081494/s1, Figure S1: FLIM images of an unstained placenta slice of the PABC case and no metastasis was observed in the placenta, Figure S2: The storage time influence on the NAD(P)H lifetime of blood samples, Figure S3: The storage time influence on the FAD lifetime of blood samples, Figure S4: NAD(P)H FLIM images of maternal blood samples showing that some cells (marked with arrows) might be WBCs.
