# Supplementary material for: Metabolic Changes in Maternal and Cord Blood in One Case of Pregnancy-Associated Breast Cancer Seen by Fluorescence Lifetime Imaging Microscopy
Source: Diagnostics (Basel). 2021 Aug 19;11(8):1494. doi: 10.3390/diagnostics11081494 (PMC8392038; doi:10.3390/diagnostics11081494)
Supplement: Supplementary file 1 [file diagnostics-11-01494-s001.zip › diagnostics-1283879-supplementary.pdf]

Supplementary Materials

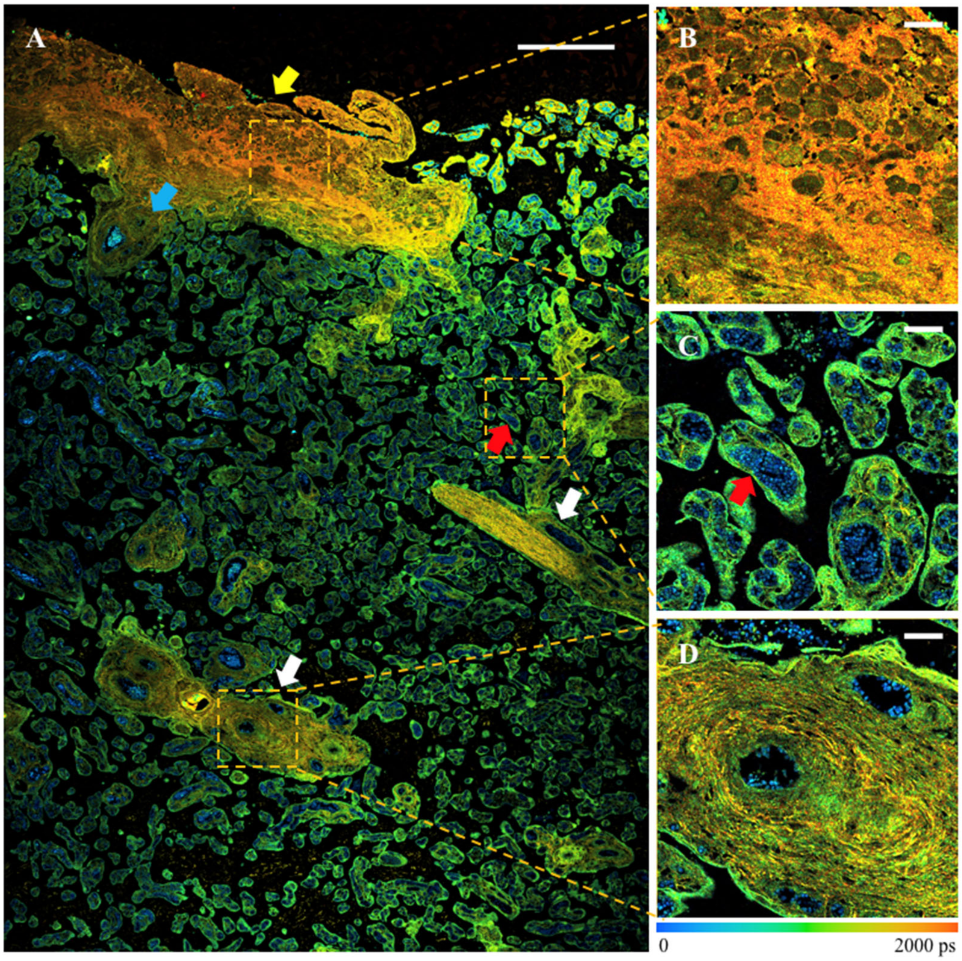

FIGURE S1. FLIM images of an unstained placenta slice of the PABC case and no metastasis was observed in the placenta. (A) The mosaic image was obtained by stitching more than 150 single FLIM images. Each single FLIM image was 1024×1024 pixels. Scale bar: 200 μm. Blue arrow: a muscular vessel. Magnified regions of yellow squares in B-D, (B) amniotic membrane (yellow arrow), (C) terminal villi (red arrow), (D) stem villi (white arrow). (B-D) Scale bar: 20 μm.

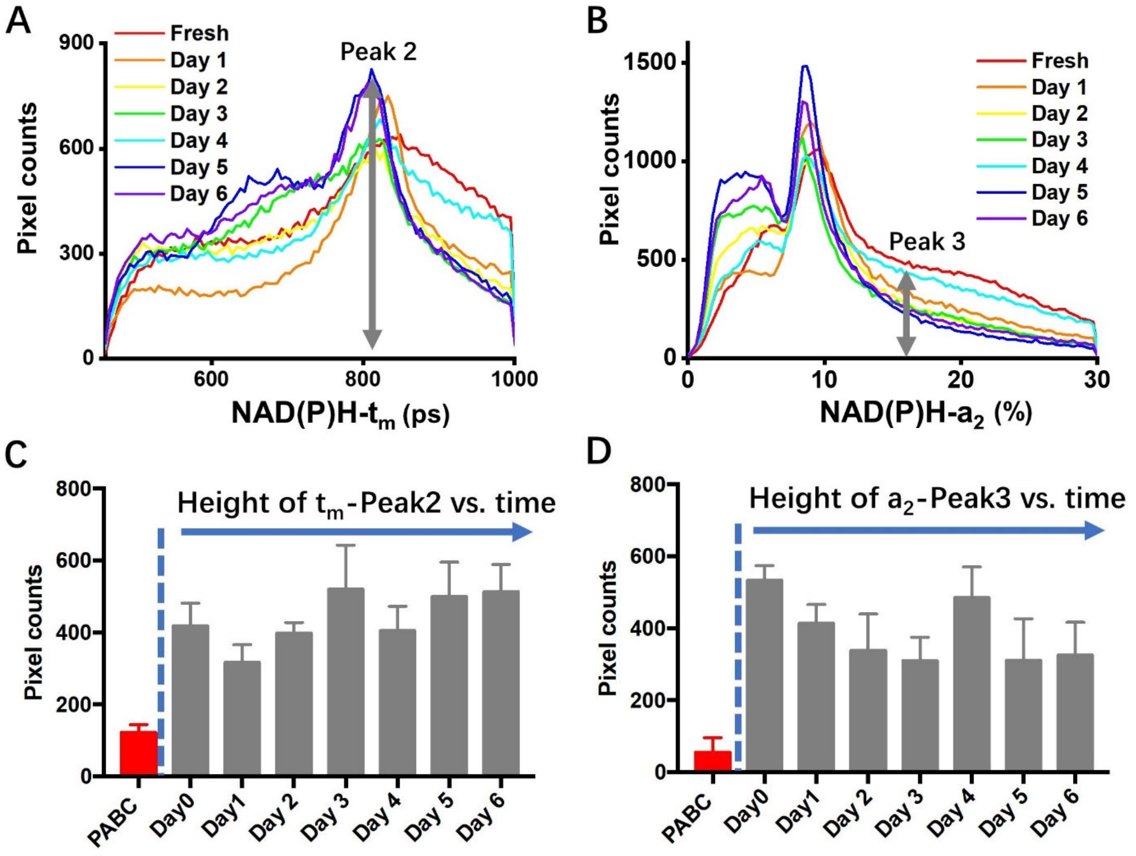

FIGURE S2. The storage time influence on the NAD(P)H lifetime of blood samples. A group of (A) t_m and (B) a_2 distribution curves of NAD(P)H FLIM images within seven days. The grey arrow in (A) is NAD(P)H-t_m Peak 2 as marked in Figure 2C, and the grey arrow in (B) is NAD(P)H-a_2 Peak 3 as marked in Figure 2F. The statistic height of peaks in A and B, as well as the other four FLIM images for each sample were averaged and displayed in C and D. The corresponding peak data of the PABC case was shown in red for comparison.

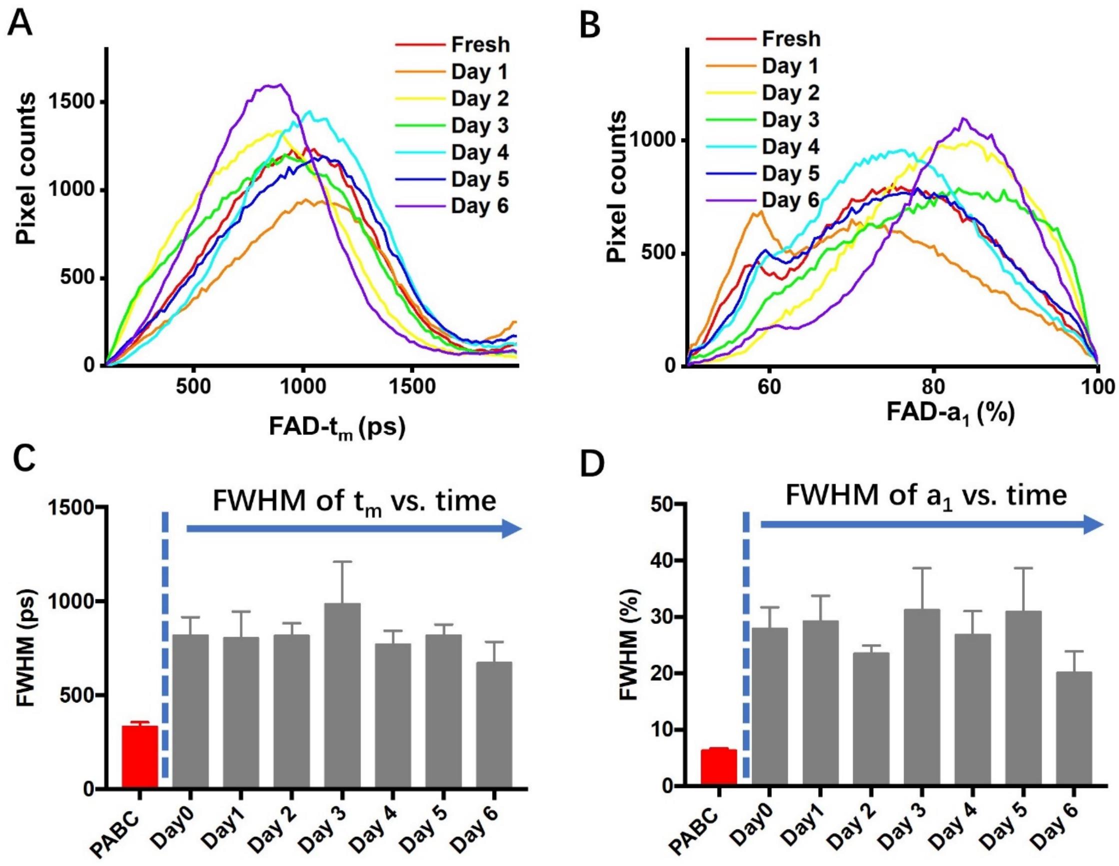

FIGURE S3. The storage time influence on the FAD lifetime of blood samples. A group of (A) t_m and (B) a_2 distribution curves of FAD FLIM images within seven days. The statistic FWHM of FAD-t_m curve in A and FAD-a_1 curve in B, as well as the other four FLIM images for each sample, were averaged and displayed in C and D with the same method in Figure 4&5. The corresponding FWHM data of the PABC case was shown in red for comparison.

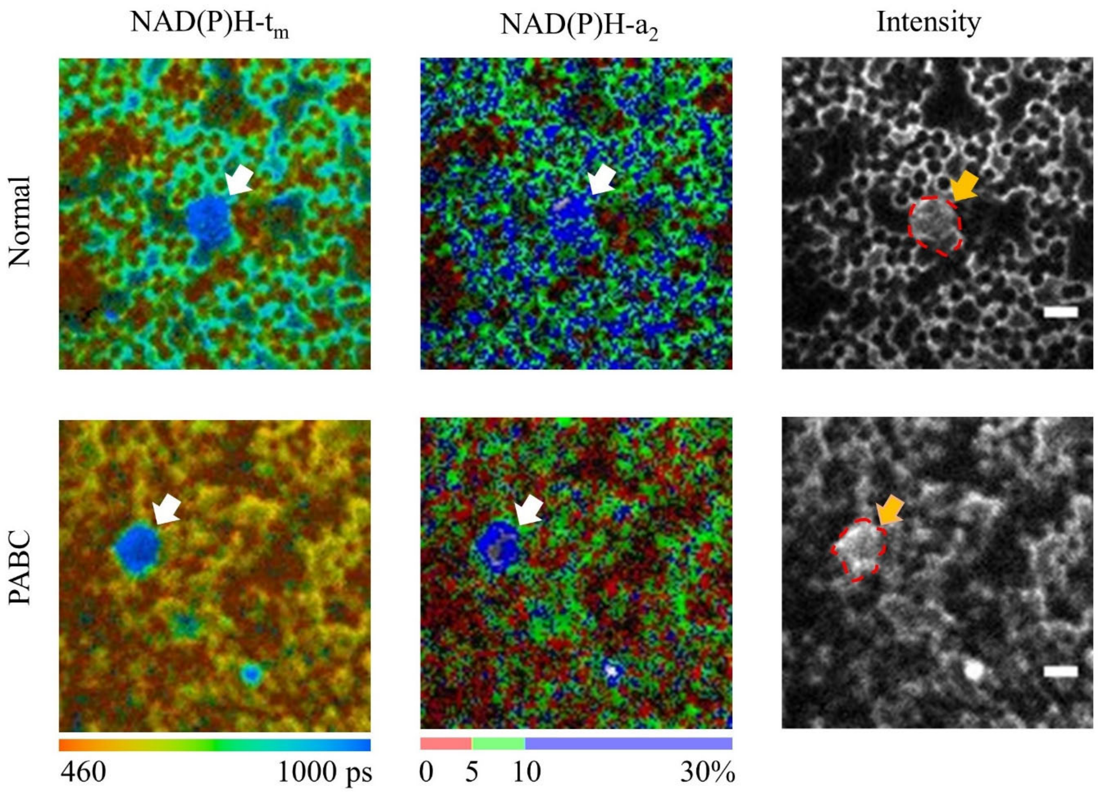

FIGURE S4. NAD(P)H FLIM images of maternal blood samples showing that some cells (marked with arrows) might be WBCs. Scale bar: 10 μm.
